# The protective immunity induced by intranasally inoculated serotype 63 chimpanzee adenovirus vector expressing human respiratory syncytial virus prefusion fusion glycoprotein in BALB/c mice

**DOI:** 10.3389/fmicb.2022.1041338

**Published:** 2022-11-18

**Authors:** Lei Huang, Mei-Qing Liu, Chang-Qing Wan, Ning-Ning Cheng, Yan-Bin Su, Yan-Peng Zheng, Xiang-Lei Peng, Jie-Mei Yu, Yuan-Hui Fu, Jin-Sheng He

**Affiliations:** College of Life Sciences and Bioengineering, Beijing Jiaotong University, Beijing, China

**Keywords:** human respiratory syncytial virus, serotype 63 chimpanzee adenoviral vector, prefusion fusion glycoprotein, intranasal immunization, immune efficacy, vaccine

## Abstract

Human respiratory syncytial virus (RSV) is a ubiquitous pediatric pathogen causing serious lower respiratory tract disease worldwide. No licensed vaccine is currently available. In this work, the coding gene for mDS-Dav1, the full-length and prefusion conformation RSV fusion glycoprotein (F), was designed by introducing the stabilized prefusion F (preF) mutations from DS-Cav1 into the encoding gene of wild-type RSV (*wt*RSV) F protein. The recombinant adenovirus encoding mDS-Cav1, rChAd63-mDS-Cav1, was constructed based on serotype 63 chimpanzee adenovirus vector and characterized *in vitro*. After immunizing mice *via* intranasal route, the rChAd63-mDS-Cav1 induced enhanced neutralizing antibody and F-specific CD8^+^ T cell responses as well as good immune protection against RSV challenge with the absence of enhanced RSV disease (ERD) in BALB/c mice. The results indicate that rChAd63-mDS-Cav1 is a promising mucosal vaccine candidate against RSV infection and warrants further development.

## Introduction

Human respiratory syncytial virus (RSV) is the leading cause of lower respiratory tract infections in infants and young children and can also cause hospitalization and death in high-risk population such as immunodeficiency patients and the elderly (Glezen et al., 1986; Nair et al., 2010). RSV accounts for about 6.7% of all children deaths from 1 month to 1 year old, ranking second after malaria (Lozano et al., 2012) and the 6.29% deaths from lower respiratory tract infections among children under the age of 5, ranking first among viral pathogens (Collaborators, 2018). Globally, there were an estimated 33.1 million children infected by RSV under 5 years old, resulting in about 3.2 million hospitalizations and 59,600 deaths (Shi et al., 2017).

RSV is a member of pneumovirus family, and is an enveloped single-stranded negative-sense RNA virus (Afonso et al., 2016). Neutralizing antibody against the fusion glycoprotein (F) is cross-protective against infection caused by RSV subtypes A and B (Graham, 2011), and is capable of reducing the disease severity and hospitalization in high-risk groups (Feltes et al., 2011). Therefore, F protein has become the main target antigen for RSV vaccine research. Moreover, a subunit vaccine candidate constructed by the stabilized prefusion RSV F (preF) is highly immunogenic, and phase 1 human immunogenicity data revealed 5- to 10-fold above baseline in neutralizing activity, which exceeded 3-fold increase in neutralization reported after experimental human challenge with RSV (Crank et al., 2019). In addition, the main protective neutralizing antibodies in human sera during natural infection are directed against the preF conformation (Blais et al., 2017; Langley et al., 2017). Interestingly, the structure-based design of prefusion-stabilized fusion protein has been confirmed by the successful application of SARS-CoV-2 vaccine (Hsieh et al., 2020). Therefore, preF is the preferred vaccine antigen.

In recent years, kinds of RSV vaccines based on preF have been developed including virus-like particle (VLP), subunit and viral vector vaccines, and many of them have entered clinical trials, (Green et al., 2015; Widjojoatmodjo et al., 2015; Green et al., 2019). In particular, human serotype Ad26 vector-based vaccine expressing preF (Ad26.RSV.preF) has been used in multiple phase 2 clinical trials including older adults (≥60 years old) and has received the Breakthrough Therapy Designation (BTD) by FDA in 2019 to prevent RSV infection in people over 60 years old. These results show that the use of recombinant adenovirus to produce RSV vaccine is one of those anticipated vaccine candidates.

Among the adenovirus exploited as vaccine vectors is chimpanzee adenovirus serotype 63 (ChAd63) due to its low infection rate in humans and high immunogenicity (Dicks et al., 2012; Afolabi et al., 2016). The malaria vector vaccine based on ChAd63 has achieved satisfactory results in phase 2 clinical trials (Rampling et al., 2018; Tiono et al., 2018), and the ChAd63-vectored HIV and HCV vaccines have also completed phase 1 clinical trials (Hartnell et al., 2018). However, neither has ChAd63 been used as a vector for developing vaccine against RSV infection nor for mucosal vaccine. In this study, the coding gene for mDS-Cav1 was obtained by introducing the stabilized preF mutations from DS-Cav1 into the encoding gene of wild-type RSV (*wt*RSV) F protein. Then, the recombinant adenovirus rChAd63-mDS-Cav1 expressing the full-length and stable preF was constructed and investigated for its potential to elicit robust immune responses by a single-dose intranasal administration in mice.

## Materials and methods

### Cells and viruses

Human embryonic kidney cells (HEK293, ATCC, Rockefeller, MD, USA) and human laryngeal carcinoma epithelial cells (HEp-2, ATCC) were cultured in Dulbecco’s Modified Eagle Medium (DMEM, HyClone Laboratories, Utah, USA) supplemented with 10% fetal bovine serum (FBS, Life Technologies Australia Pty Ltd., NSW, Australia), L-glutamine (2 mol/l), penicillin G (40 U/ml) and streptomycin (100 μg/ml) in a humidified incubator at 37°C, 5% CO_2_. A E1/E3-deleted, replication-deficient recombinant adenoviral vectors based on ChAd63 were constructed using DNA Assembly kit (New England Biolabs, Ipswich, MA, USA). To obtain the mDS-Cav1-coding sequence, the stabilized preF mutations from DS-Cav1 were introduced into the *wt*RSV F gene (McLellan et al., 2013). rChAd63-mDS-Cav1 expressing mDS-Cav1 was constructed as described previously (Zou et al., 2018).

To prepare and purify rChAd63-mDS-Cav1, HEK293 cells were infected with rChAd63-mDS-Cav1 at 10 MOI and 37°C. At 60–72 h post infection, cells were collected by centrifugation with 4000 rpm at 4°C for 10 min, resuspended in 0.1 mol/L Tris–HCl (pH 8.0) and lysed with sodium deoxycholate. Then, DNAase I was added to cell lysate for incubation 1 h at 37°C and mixing every 10 min, and the supernatants were collected at 3000 *g* for 15 min at 4°C and layered on top of a discontinuous CsCl gradient composed of 3.0 ml of 1.35 g/ml CsCl and 3.0 ml of 1.25 g/ml CsCl. The gradients were ultracentrifuged at 35000 rpm, 4°C for 1 h in a P40ST rotor (Hitachi, Japan). The virus band at the interface of 1.25 g/ml and 1.35 g/ml CsCl was collected and added to ultracentrifuge tube, and ultracentrifuged at 35000 rpm, 4°C for 16 h in the P55ST rotor (Hitachi). The virus band was collected and dialyzed with Slide-A-Lyzer (Thermo Fisher Scientific, Waltham, MA, USA) in 0.01 mol/L Tris–HCl for 24 h at 4°C. After purification, the titers of the purified rChAd63/mDS-Cav1 were measured by Qubit fluorometric quantitation (Thermo Fisher Scientific) method and Adeno-X rapid titer (Clontech, Mountain View, CA, USA) method, and the expression of mDS-Cav1 was detected by Dot blot.

The *wt*RSV of subgroup A strain Long (ATCC) was propagated in HEp-2 cells in DMEM supplemented with 2% FBS, 2 mM L-glutamine and 1% P/S in a humidified incubator at 37°C, 5% CO_2_. Formalin inactivated-RSV (FI-RSV) was prepared as described previously (Kim et al., 1969; Mapletoft et al., 2008).

### *In vitro* expression and characterization of RSV preF encoded by rChAd63-mDS-Cav1

HEK-293 cells were infected with rChAd63-mDS-Cav1. At 48 h after infection, the cells were collected, washed twice with cold PBS, and blotted onto nitrocellulose membranes. The antibodies of palivizumab, 131-2A and D25 specific for epitopes of site II, site I and site Ø in F, respectively, were constructed and prepared by cloning the genes encoding heavy (VH) and light (VL) chain, respectively, into expression vectors, and were employed to incubation with the above samples. After further incubated with IRDye 800CW goat anti-human IgG or anti-mouse IgG (Li-Cor Biosciences, Lincoln, NE, USA), these samples were finally visualized by the LI-COR Odyssey Clx Imager system (Li-Cor Biosciences).

### Animals, immunization, and virus challenge

Specific pathogen-free (SPF) female BALB/c mice, aged 6–8 weeks, were purchased from Beijing Vital River Laboratory Animal Technology Co., Ltd. and kept under SPF conditions. BALB/c mice were lightly anesthetized with Avertin and immunized either with rChAd63-mDS-Cav1, rChAd63-empty (1 × 10^10^ VP/mouse) and *wt*RSV-Long (1 × 10^5^ PFU/mouse) in 50 μl intranasally, respectively, or with FI-RSV (1.25 × 10^6^ PFU/mouse) in 50 μl intramuscularly. On 28^th^ day after immunization, BALB/c mice were challenged intranasally with *wt*RSV-Long strain (1 × 10^6^ PFU/mouse) in 50 μl.

### Humoral immunity against RSV preF and postfusion F (postF) analyzed by ELISA

For bronchoalveolar lavage fluids (BALF), the mice were first anesthetized by inhalation of isoflurane and sacrificed and BALF were obtained by lavage with three successive 1 ml volumes of PBS from cannulation of the trachea.

preF (Krarup et al., 2015), and postF proteins were expressed by baculovirus expression system. The cells and culture supernatant were separately harvested 3 days post infection. preF and postF were purified with nickel affinity chromatography (Ni-NTA agarose, Thermo Fisher Scientific), respectively.

The ELISA method was used to detect titers of serum IgG and IgA antibody against preF or postF. Briefly, 96-well microtiter plates were coated with purified preF or postF proteins overnight at 4°C and blocked with 5% BSA in PBS for 2 h at 37°C, and BALF and serially diluted sera were added to the plates. The antibody titers were detected by HRP-conjugated anti-mouse IgG or IgA (Santa Cruz Biotechnology, Santa Cruz, CA, USA) and the wells were developed with soluble TMB substrate (Sigma, St. Louis, MO, USA). The reaction was stopped with 50 μl 2 M H_2_SO_4_, and analyzed at 450 nm using an ELISA plate reader (Tecan, Grödig, Austria).

### Serum preF-specific IgG subclass determined by ELISA

To analyze the Th1/Th2 skewing after immunization, RSV-specific antibody subclasses were measured by ELISA. After 96-well microtiter plates coated with purified preF, serially diluted sera were added to the plates, and preF-specific IgG1 or IgG2a were detected using HRP-conjugated anti-mouse IgG1 or IgG2a and analyzed at 450 nm using an ELISA plate reader (Tecan). The titers of preF-specific IgG1 and IgG2a antibodies were defined as the reciprocal of the serum dilution that gave an OD450 value of 2.1-fold over naïve sera.

### RSV-specific neutralizing antibody assay

Neutralizing antibodies against *wt*RSV-Long were determined by the method described previously (Ma et al., 2021). Serially diluted sera were mixed with RSV-mGFP and incubated for 1 h at 37°C, then the mixtures were added to 96-well plates with HEp-2 cells. After incubation for 48 h at 37°C, the fluorescence intensity was detected using a microplate reader (Molecular Devices, Silicon Valley, CA, USA) with excitation wavelength of 479 nm and emission wavelength of 517 nm. Neutralizing antibody titers were calculated as the antibody concentration that caused a 50% reduction in fluorescence intensity (IC50).

### Enzyme-linked immunospot assay (ELISpot)

The ELISPOT assay was used to analyze the F-specific CD8+ T-cell response. At the 21^st^ day after a single intranasal immunization with recombinant adenovirus, the splenocytes were collected and stimulated with RSV F protein H-2 K^d^ restricted CTL epitopes (KYKNAVTEL and TYMLTNSEL, respectively; purity ≥95%) for 24 h, and the number of F–specific IFN-γ-secreting CTL cells in the spleen were counted using a ELISPOT reader (BD Biosciences, Franklin Lakes, NJ, USA).

### RSV titer in lungs

Three weeks after the immunization, the mice were challenged intranasally with 1 × 10^6^ PFU *wt*RSV-Long strain, and sacrificed on day 5 after challenge. The lungs from mice were harvested, weighed, homogenized, and RSV titers in the supernatants were measured by RT-qPCR as described previously (Jiao et al., 2017) and normalized by the concentration of total lung tissue RNA and the weight of total lung.

### Lung histopathology

Mice were sacrificed on day 5 after challenge, and the lungs from mice were harvested and fixed in formaldehyde. Four-micrometer-thick sections were stained with hematoxylin and eosin (H&E). The lung pathology of RSV-challenged mice was evaluated as described previously (Jiao et al., 2017).

### Statistical analysis

Statistical analysis was performed with SPSS21 software (SPSS, Chicago, IL, USA), and the means of multiple groups were compared by one-way analysis of variance. *p* < 0.05 was considered significant.

## Results

### *In vitro* characterization of the expressed preF by rChAd63-mDS-Cav1

**Figure 1:**
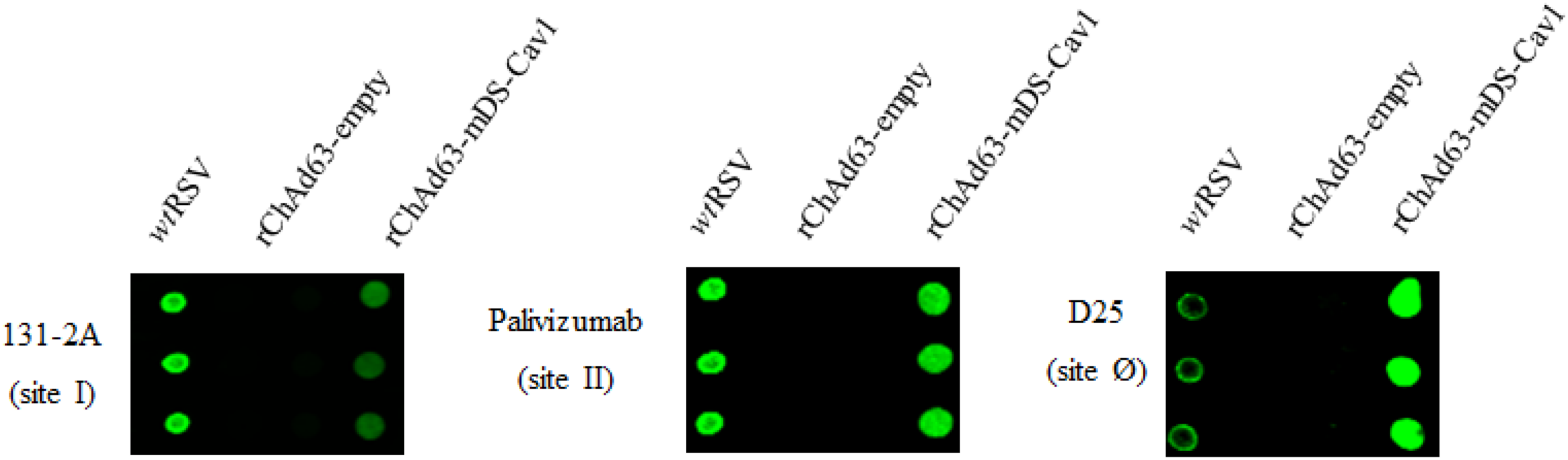
*In vitro* characterizations of the encoded preF from rChAd63-mDS-Cav1. After 48  h following infection with rChAd63-mDS-Cav1, HEK293 cells were collected and Dot blot assay was performed. The prefusion epitope Ø in the expressed preF by rChAd63-mDS-Cav1 was detected readily.

Antigenic site I is mainly present on postF, and antigenic site II is present on preF and postF, and antigenic site zero (Ø) is specific to the preF (Mousa et al., 2017; Patel et al., 2019). To identify the characteristics of preF encoded by rChAd63-mDS-Cav1 *in vitro*, the expressed preF by rChAd63-mDS-Cav1 was examined by Dot Blot using antibodies specific to total F (palivizumab, site II-specific mAb), postF (131-2A, site I-specific mAb) and preF (D25, site Ø -specific mAb; Figure 1). Compared with the signal intense produced by 3 kinds of different antibodies, the preFs from HEK293 cells infected with rChAd63-mDS-Cav1 were recognized from the best to the worst by D25, Palivizumab and 131-2A, respectively, which indicated the encoded preF by rChAd63-mDS-Cav1 predominantly displayed the prefusion conformation.

### preF-specific systemic and mucosal antibody responses induced by a single immunization with rChAd63-mDS-Cav1

**Figure 2:**
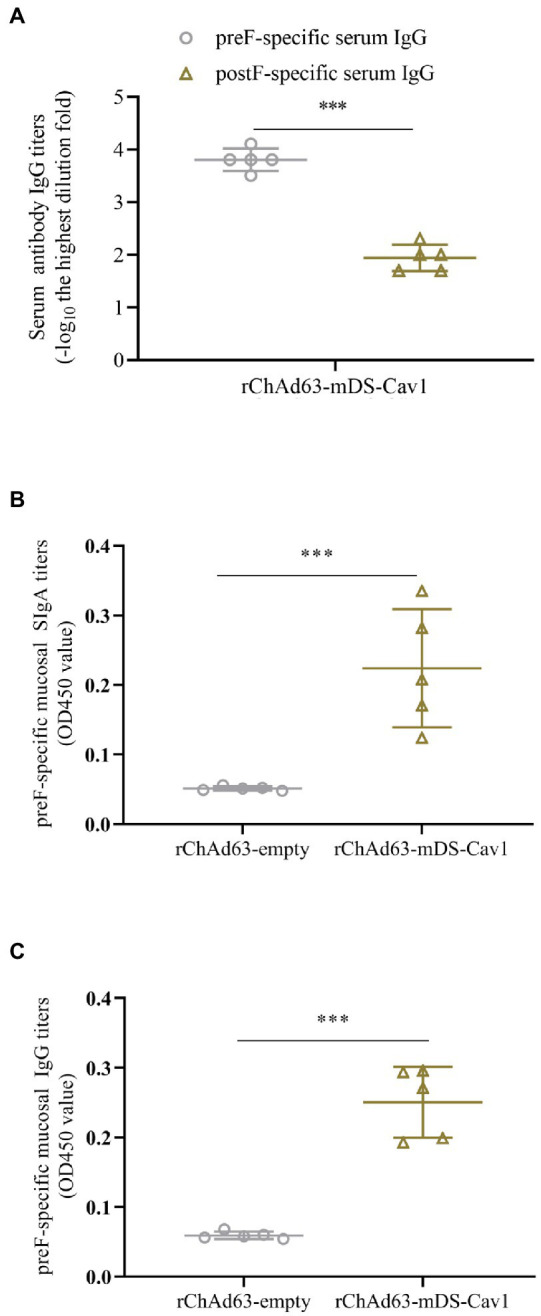
preF-specific serum and mucosal antibody responses in BALB/c mice following immunizations. BALB/c mice (*n* = 5) were immunized intranasally with recombinant adenovirus (1 × 10^10^ VP/mouse). On the 21^st^ day after immunization, the preF-specific IgG and IgA were detected by ELISA. Serum anti-preF IgG and anti-postF IgG **(A)**, mucosal anti-preF SIgA **(B)** and IgG **(C)** responses induced by rChAd63-mDS-Cav1. Data were shown as mean ± SD, *** *p* < 0.001.

To determine the ability of rChAd63-mDS-Cav1 to induce preF-specific antibody response, BALB/c mice were intranasally immunized with rChAd63-mDS-Cav1and rChAd63-empty (1 × 10^10^ VP/mouse), respectively, and antibodies against RSV preF and postF were measured by ELISA (Figure 2A). The results showed that the rChAd63-mDS-Cav1 produced significant preF-specific IgG responses, but not postF. The BALF were obtained by lavage with three successive 1 ml volumes of PBS from cannulation of the trachea. After centrifugation (1,000 *g* for 5 min), mucosal SIgA and IgG responses were analyzed by ELISA, respectively. As shown in Figures 2B,C, BALB mice following immunization with those mice by rChAd63-mDS-Cav1 generated mucosal SIgA and IgG responses compared with those mice by rChAd63-empty immunization (*p* < 0.05).

### The high titer of neutralizing antibody evoked by a single immunization with rChAd63-mDS-Cav1

**Figure 3:**
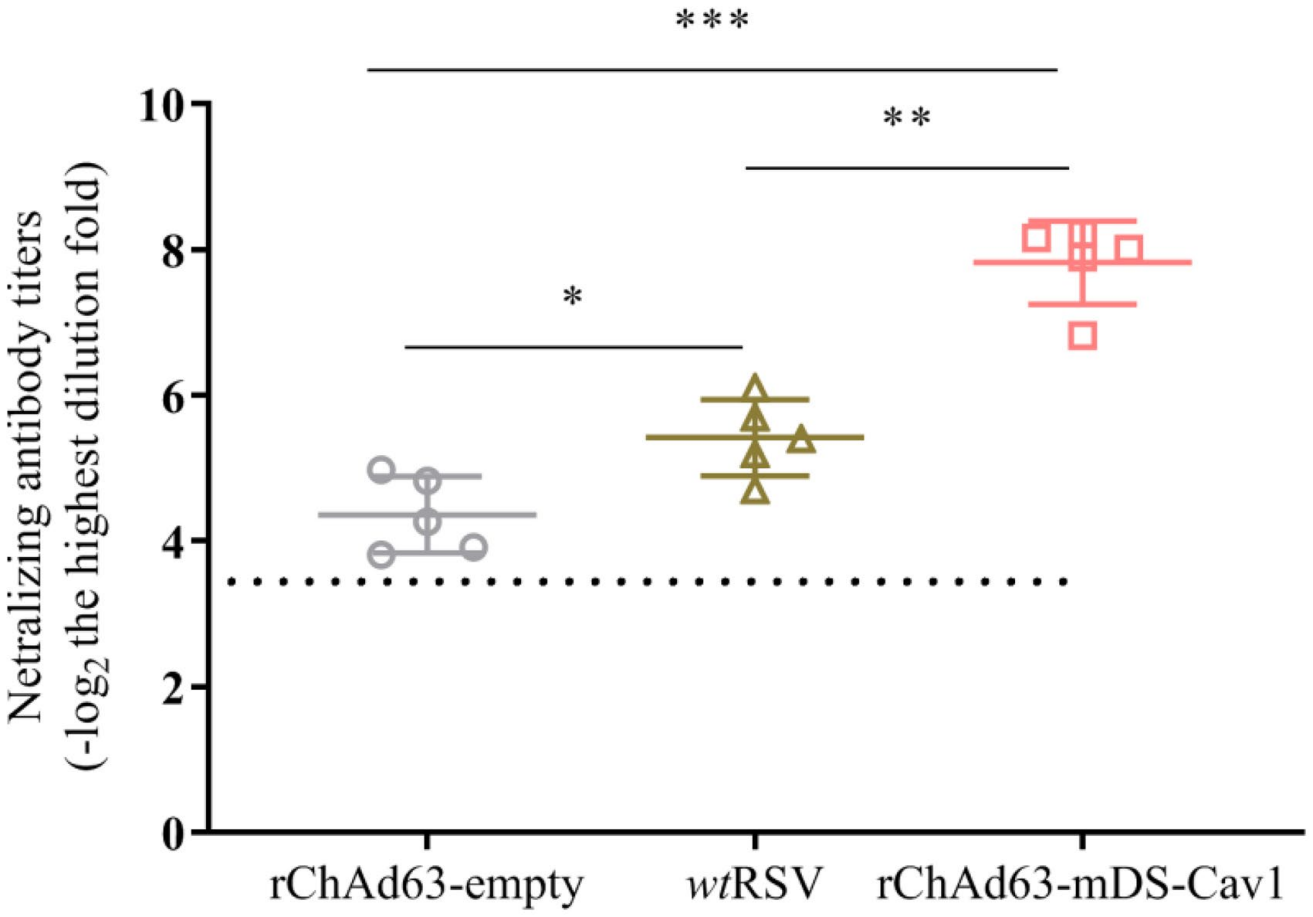
Serum neutralizing antibody responses following immunization with rChAd63-mDS-Cav1 in BALB/c mice. BALB/c mice (*n* = 5) were immunized intranasally with recombinant adenovirus (1 × 10^10^ VP/mouse) or *wt*RSV (1 × 10^5^ PFU/mouse), and serum neutralizing antibody titers were analyzed on day 21 after immunization. Data were shown as mean ± SD, *** *p* < 0.001.

Neutralizing antibody against RSV F is the critical factor to prevent RSV infection. We therefore tested the induced serum neutralizing antibody titers by RSV-mGFP-based method (Figure 3). The neutralizing antibody results showed that, compared with the BALB mice immunized by either rChAd63-empty or *wt*RSV-Long, the mice experiencing a single intranasal vaccination with rChAd63-mDS-Cav1 could generate higher titer of neutralizing antibody (**** *p* < 0.0001).

### A balanced or mixed Th1/Th2 immune response invoked following immunization with rChAd63-mDS-Cav1 in BALB/c mice

**Figure 4:**
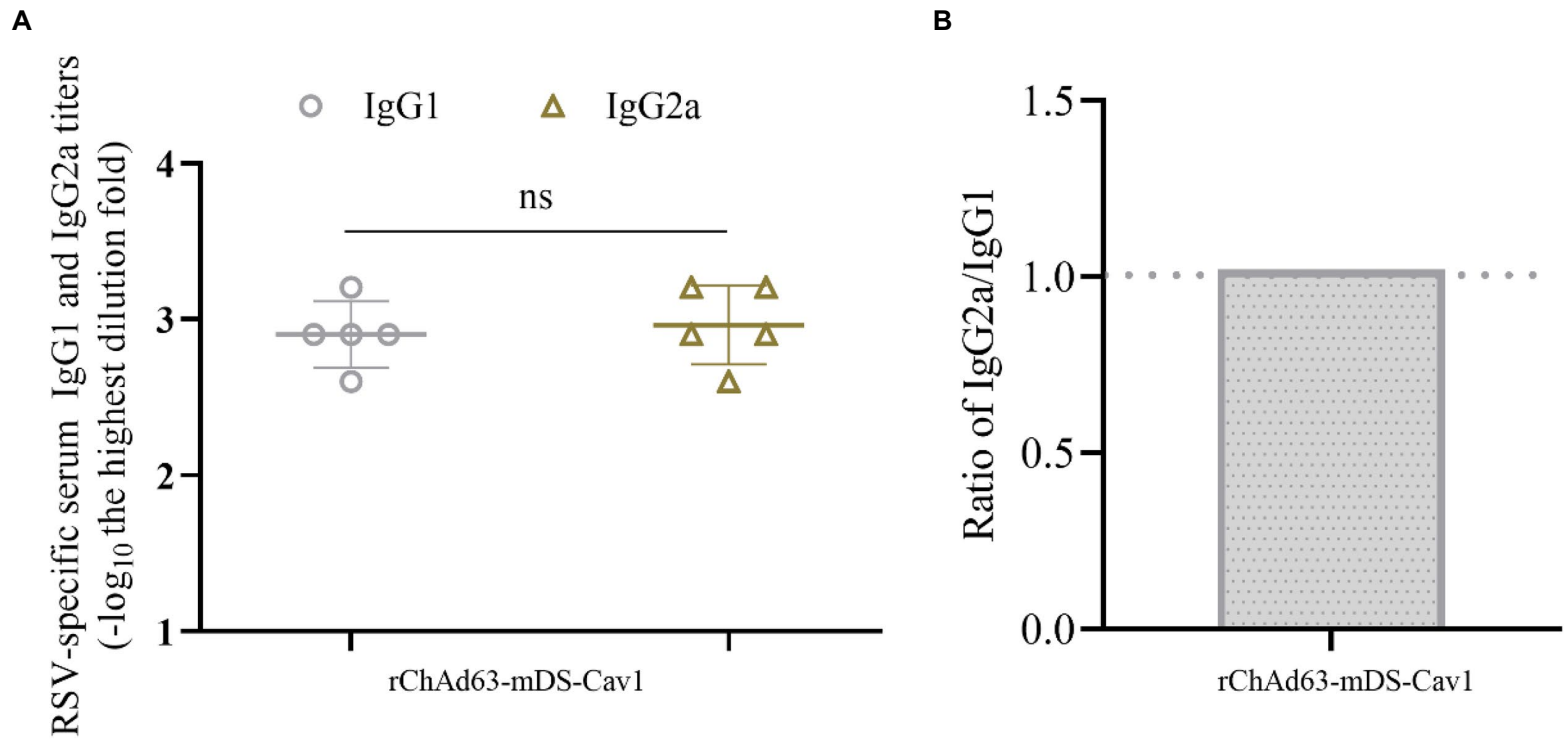
Serum anti-RSV isotype response following immunization with rChAd63-mDS-Cav1 in BALB/c mice. BALB/c mice (*n* = 5) were immunized intranasally with rChAd63-mDS-Cav1, and the serum anti-RSV isotype responses were detected by ELISA. **(A)** The titers of anti-RSV IgG1 and IgG2a. The antibody titers were defined as the reciprocal of the highest serum dilution that gave an OD450 value of 2.1-fold over naïve sera. **(B)** The ratio of IgG2a and IgG1 titers. Data were shown as mean ± SD, ns: no statistical difference.

To analyze the Th1/Th2 skewing, the preF-specific serum antibody IgG1 and IgG2a were detected by ELISA (Figure 4). The titers of preF-specific serum antibodies IgG1 and IgG2a induced by rChAd63-mDS-Cav1 were not statistically different, and the IgG2a/IgG1 ratio was close to 1, indicating that rChAd63-mDS-Cav1 stimulated balanced or mixed Th1/Th2 immune responses beneficial to avoid the enhanced respiratory disease (ERD) following the vaccination of FI-RSV.

### F-specific CD8^+^ T-cell immune responses instigated by immunization with rChAd63-mDS-Cav1 in BALB/c mice

**Figure 5:**
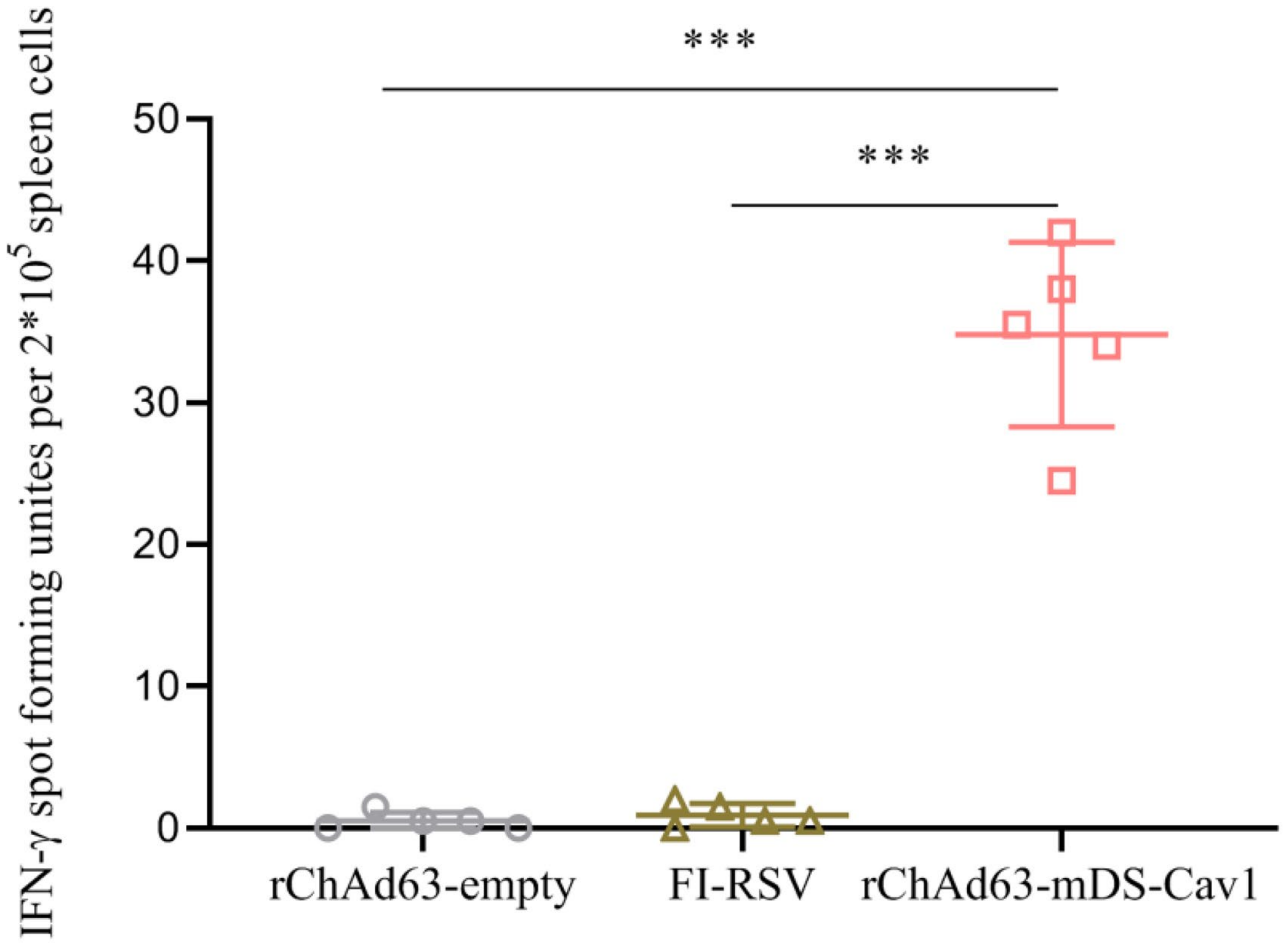
Specific CD8+ T cell responses after immunization with rChAd63-mDS-Cav1. BALB/c mice (*n* = 5) were immunized intranasally with rChAd63-mDS-Cav1, and RSV F-specific CD8+ T cell responses were detected by ELISpot on the 21st day after immunization. Data were shown as mean ± SD, *** *p* < 0.001.

It has been widely accepted that CD8+ T-cell responses specific for virus antigens are very important to eliminate intracellular viruses, and reduce the disease severity. Therefore, the cellular immunity was assessed by isolating splenocytes at 21 days after immunization. After splenocytes stimulated with RSV FH-2 K^d^ restricted peptides, we used ELISpot to detect the number of RSV F-specific CD8^+^ T cells secreting IFN-γ (Figure 5). Compared with rChAd63-empty, a significantly increased RSV F-specific CD8+ T cells secreting IFN-γ were observed in rChAd63-mDS-Cav1-immunized mice (**** *p* < 0.0001). The results showed that single intranasal vaccination with rChAd63-mDS-Cav1 induced strong CD8^+^ T cell responses.

### Protective efficacy and safety following immunization with rChAd63-mDS-Cav1 and challenge with *wt*RSV in mice

**Figure 6:**
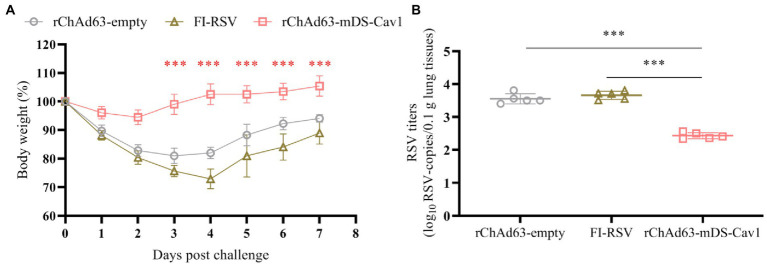
The losses of body weight and virus titers in lungs from BALB/c mice after RSV challenge.The BALB/c mice (*n* = 5) were challenged intranasally with *wt*RSV-Long strain (1 × 10^6^ PFU/mouse) at day 28 after the immunization. **(A)** The losses of body weight were observed daily for 7 consecutive days in which 100% body weight was set at day 0 when the mice were challenged. **(B)** Lung RSV titer were measured by RT-qPCR at day 5 after the challenge. Data were shown as mean ± SD, *** *p* < 0.001.

The important parameters to evaluate the protective effect of RSV vaccine candidates are the weight changes of mice and the virus titers of lung tissue after challenge. On the 28^th^ day after immunization, *wt*RSV-Long viruses at 1 × 10^6^ PFU/mouse were used for the challenge experiment, and the weight changes of the mice were monitored daily for 7 consecutive days in which 100% body weight was set at day 0 when the mice were challenged (Figure 6A). The body weight loss reached 20% in mice immunized with FI-RSV or rChAd63-empty, which contrasted with less than 10% in mice immunized with rChAd63-mDS-Cav1. Compared with FI-RSV and rChAd63-empty, immunization with rChAd63-mDS-Cav1 could reduce weight loss of RSV-challenged mice (*p* < 0.001).

On the 5^th^ day after challenge, the viral copy numbers in lung tissues were detected by RT-qPCR, calculated according to the standard curve, and normalized by the concentration of total lung tissue RNA and the weight of total lung (Figure 6B). RT-qPCR results showed that compared with FI-RSV and the rChAd63-empty, rChAd63-mDS-Cav1 significantly decreased the virus titer of lung tissue (*p* < 0.01), and effectively prevent RSV infection. The 16.8- or 13.2-fold reduced lung viral copy numbers were achieved by application of rChAd63-mDS-Cav1, compared to FI-RSV or rChAd63-empty. The above results indicated that immunization of mice with rChAd63-mDS-Cav1 can lead to effective immune protection.

**Figure 7:**
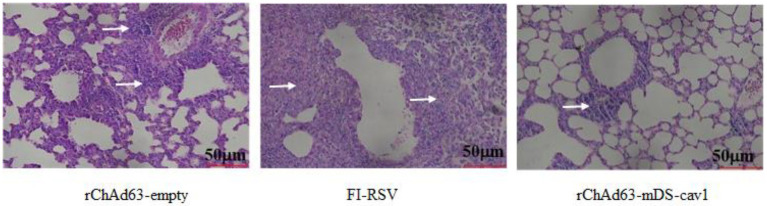
Lung histopathology at day 5 after *wt*RSV-challenge. The immunized mice were challenged intranasally with *wt*RSV-Long strain (1 × 10^6^ PFU/mouse) at day 28 after the immunization. The lung tissues were collected at day 5 post RSV challenge and prepared for analyses of pulmonary histopathology by H&E stain.

The histopathological changes in lung tissue of immunized mice on the 5^th^ day after RSV challenge were examined by hematoxylin and eosin staining (Figure 7). Compared with FI-RSV and rChAd63-empty, the lung tissues from mice immunized with rChAd63-mDS-Cav1 displayed clear and complete alveolar structure, absence of alveolar collapses and the less inflammatory cell infiltration around blood vessels and bronchi. These results suggested that the recombinant adenovirus vector vaccine rChAd63-mDS-Cav1 exhibited better protection, and importantly, the expected safety with the absence of ERD.

## Discussion

Since the knowledge and understating of the pre-fusion conformation stabilized preF and neutralization-sensitive viral epitopes, the development of RSV vaccine has advanced sharply. Among them, several virus-vectored RSV vaccine candidates have been successfully developed upon DS-Cav1(Phan et al., 2017; Liang et al., 2020). In this study, we designed the full-length and transmembrane version of DS-Cav1 based on *wt*RSV F protein, whereby, constructed rChAd63-mDS-Cav1 and investigated the immune efficacy and safety in BALB/c mice. After single intranasal immunization, the rChAd63-mDS-Cav1 could induce enhanced neutralizing antibody and F-specific CD8+ T cell responses as well as effective immune protection against RSV infection with the absence of ERD in BALB/c mice.

So far, there have four RSV vaccine candidates of Ad26.RSV.preF, MVA-BN-RSV, RSVpreF and MV-012–968 that displayed good safety, immunogenicity and immune efficacy following intramuscular inoculation and human viral challenge (HVC) studies in adults and Phase 2 trials (Sadoff et al., 2022; Schmoele-Thoma et al., 2022). Except for MV-012–968, all the other three vaccine candidates have received BTD and progressed to phase 3 trials for the prevention of RSV infection in population over 60 years of age. Recently, GSK stops phase 3 trials of maternal RSV vaccine with adjuvant in pregnant women, RSVpreF3, after seeing safety signal, but the vaccines of RSVpreF3 OA from GSK for older adults and RSVpreF from Pfizer for elderly and maternal population have achieved positive pivotal phase 3 data. Additionally, vaccine candidates delivered as mRNA, mRNA-1,345, or *via* intranasal route, MV-012-968 and VAD00001, have been in their phase 2 trials.

Although there are still many reports about Ad5-vectored vaccines, the high level of neutralizing antibody against Ad5, pre-existing in the circulation of majority population from the developing countries, affects its application as vaccine vector *via* intramuscular route (Xiang et al., 2006; Mast et al., 2010; Sun et al., 2011; Zak et al., 2012; Sasso et al., 2020; Jeyanathan et al., 2022). Compared with intramuscular route, generally, intranasal inoculation of Ad5-vectored vaccine can reduce this kind of interference to some extent. However, the higher immune response and immune efficacy against SARS-CoV-2 have been observed in mice vaccinated intranasally by chimpanzee Ad vectored vaccine other than Ad5 vectored vaccine (Afkhami et al., 2022).

As we know, Ad26 is same human adenovirus as Ad5, but it has lower titers of vector-specific neutralizing antibodies worldwide (Barouch et al., 2011) whereby Ad26.RSV.preF has been developed, and a single immunization has achieved 5.8-fold and 2.4–3.2-fold neutralizing antibody titers against RSV above baselines in health adults aged 18–50 years and ≥ 60 years (Williams et al., 2020; Sadoff et al., 2021), respectively. Although Ad26-based RSV vaccine displays good safety and immunogenicity, it should be noted the immunogenicity was a little bit weaker in older adults of ≥60 years than ≤50 years, and was also less suitable for mucosal vaccination because of the primary receptor usage of CD46 (Lapuente et al., 2021). Meanwhile, the very rare but serious cases of thrombosis with thrombocytopenia syndrome (TTS) has been reported after vaccination with COVID-19 non-replicant adenovirus vector-based vaccines, which is likely related to the direct activation of platelets and endothelia by the adenovirus vector or the induced autoimmunity by the formed complex of adenovirus/platelet factor 4 (PF4) following accidental intravenous introduction of adenovirus, however, respiratory mucosal delivery of Ad-vectored vaccines may avert this adverse outcome (Baker et al., 2021; Tsilingiris et al., 2021; Afkhami et al., 2022).

Similar as the advantage of other ChAd vectors, there is little pre-existing immunity in humans against ChAd63 viruses, and ChAd63 vectored vaccines also displays potent immunogenicity and the feasibility for mucosal immunization (Sasso et al., 2020; Lapuente et al., 2021). Therefore, we think it is reasonable to choose ChAd63 as the potential vector to develop the next generation of RSV vaccine.

At this proof of concept study, we constructed rChAd63-mDS-Cav1 to express the modified preF based on the DS-Cav1, the prototype of preF. In this study, the challenge results after a single intranasal immunization of rChAd63-mDS-Cav1 showed that both preF-specific antibody responses in sera and respiratory mucosa and F-specific CD8+ T-cell responses are induced, and both preF-specific IgG and SIgA antibody response can significantly reduce the RSV virus titer in the lungs, as well as prevent weight loss. Therefore, we think that ChAd63 is qualified as mucosal vaccination vector and can induce good immune protection when preF expressed.

Previous studies have shown that in infants and young children, the level of CD8^+^ T cells is closely related to the severity of RSV disease (Larranaga et al., 2009). In children with cellular immune deficiency, the RSV virus replication time is prolonged and the disease severity increases (Fishaut et al., 1980), indicating that cellular immunity plays an important role in clearing intracellular infections and inhibiting lung pathology (Schmidt et al., 2018). Therefore, an ideal RSV vaccine should be able to induce the production of high-titer neutralizing antibodies and cellular immunity at the same time (Schmidt et al., 2020). The results of this study showed that the recombinant adenoviruses carrying preF gene produced good CD8^+^ T cell immune responses, indicating that ChAd63-vectored RSV vaccine has achieved the expected effect.

In addition, the main concern on the safety of RSV vaccine is the potential immunopathogenesis or ERD induced by the vaccine candidate. The data of lung pathology on mice 5 days after challenge showed that the lung pathological damage of rChAd63-mDS-Cav1 was remarkably reduced compared with those observed in the vector control group, especially the FI-RSV group. The IgG1/IgG2a ratio produced by rChAd63-mDS-Cav1 immunized mice is close to 1, which indicates that rChAd63-mDS-Cav1 induced a mixed Th1/Th2 cytokine immune response capable of avoiding ERD. Therefore, the vaccine has good safety and the immunized mice risk little to develop ERD after being infected with RSV.

In short, the constructed rChAd63 vector has been successfully applied to the development of RSV vaccine. The rChAd63-mDS-Cav1 expressing one full-length and transmembrane version of pre-Fs was highly effective and safe against RSV challenge in BALB/c mice after a single intranasal vaccination, suggesting that rChAd63 has the potential used to develop next generation of RSV vaccine.

## Data availability statement

The raw data supporting the conclusions of this article will be made available by the authors, without undue reservation.

## Ethics statement

The animal study was reviewed and approved by Beijing Laboratory Animal Research Center.

## Author contributions

LH and M-QL: conceptualization, methodology, software, formal analysis, writing - original draft. C-QW: visualization, methodology, software, investigation. N-NC: resources, supervision. Y-BS: software, validation. Y-PZ: writing – review and editing. X-LP: resources. J-MY: software. Y-HF: and J-SH: conceptualization, funding acquisition, resources, supervision, writing – review and editing. All authors contributed to the article and approved the submitted version.

## Funding

This work was supported by the National Natural Science Foundation of China (32070922).

## Conflict of interest

The authors declare that the research was conducted in the absence of any commercial or financial relationships that could be construed as a potential conflict of interest.

## Publisher’s note

All claims expressed in this article are solely those of the authors and do not necessarily represent those of their affiliated organizations, or those of the publisher, the editors and the reviewers. Any product that may be evaluated in this article, or claim that may be made by its manufacturer, is not guaranteed or endorsed by the publisher.
